# Variations in survival after cardiac arrest among academic medical center-affiliated hospitals

**DOI:** 10.1371/journal.pone.0178793

**Published:** 2017-06-05

**Authors:** Michael Christopher Kurz, John P. Donnelly, Henry E. Wang

**Affiliations:** 1 Department of Emergency Medicine, University of Alabama School of Medicine, Birmingham, Alabama, United States of America; 2 Division of Preventive Medicine, Department of Medicine, University of Alabama School of Medicine, Birmingham, Alabama, United States of America; 3 Department of Epidemiology, School of Public Health, University of Alabama at Birmingham, Birmingham, Alabama, United States of America; Stanford University School of Medicine, UNITED STATES

## Abstract

**Background:**

Variation exists in cardiac arrest (CA) survival among institutions. We sought to determine institutional-level characteristics of academic medical centers (AMCs) associated with CA survival.

**Methods:**

We examined discharge data from AMCs participating with Vizient clinical database–resource manager. We identified cases using ICD-9 diagnosis code 427.5 (CA) or procedure code 99.60 (CPR). We estimated hospital-specific risk-standardized survival rates (RSSRs) using mixed effects logistic regression, adjusting for individual mortality risk. Institutional and community characteristics of AMCs with higher than average survival were compared with those with lower survival.

**Results:**

We analyzed data on 3,686,296 discharges in 2012, of which 33,700 (0.91%) included a CA diagnosis. Overall survival was 42.3% (95% CI 41.8–42.9) with median institutional RSSR of 42.6% (IQR 35.7–51.0; Min-Max 19.4–101.6). We identified 28 AMCs with above average survival (median RSSR 61.8%) and 20 AMCs with below average survival (median RSSR 26.8%). Compared to AMCs with below average survival, those with high CA survival had higher CA volume (median 262 vs.119 discharges, p = 0.002), total beds (722 vs. 452, p = 0.02), and annual surgical volume (24,939 vs. 13,109, p<0.001), more likely to offer cardiac catheterization (100% vs. 72%, p = 0.007) or cardiac surgery (93% vs. 61%, p = 0.02) and cared for catchment areas with higher household income ($61,922 vs. $49,104, p = 0.004) and lower poverty rates (14.6% vs. 17.3%, p = 0.03).

**Conclusion:**

Using discharge data from Vizient, we showed AMCs with higher CA and surgical case volume, cardiac catheterization and cardiac surgery facilities, and catchment areas with higher socioeconomic status had higher risk-standardized CA survival.

## Background

In-hospital cardiac arrest (IHCA) is an important issue for institutions caring for the sick and injured. In the United States, an estimated 209,000 IHCA may occur annually.[1] Mortality from IHCA is significant, ranging from 58 to 100% with only small improvements in the last quarter century.[2, 3] In addition, it varies widely between institutions and has been associated with institutional size, geographic location, and teaching status. [4]^,^ [5]

Unlike out-of-hospital cardiac arrest, which is largely a random event, IHCA is often a predictable event where progressive deterioration in clinical status leads to respiratory failure and/or cardiogenic shock. Despite the publication of clinical care guidelines for primary and secondary prevention of cardiac arrest, [6] [7] wide variations persist in institutional survival, suggesting potential associations with additional institutional characteristics.

Using data from Vizient (formerly the University HealthSystem Consortium) clinical database–resource manager [CDB/RM], we sought to evaluate hospital variations in cardiac arrest survival and identify institutional characteristics that distinguish between best and weakest performers.

## Methods

### Study design

In this investigation, we conducted a retrospective cohort analysis of hospital discharge data from Vizient’s clinical database–resource manager (CDB/RM) linked with the American Hospital Association (AHA) institutional database and the American Community Survey (ACS). The study was approved by the Institutional Review Board of the University of Alabama at Birmingham.

### Study setting

Vizient includes, but is not now limited to, a consortium of approximately 300 hospitals across 120 academic health systems in the United States. Vizient CBD/RM members share performance data to facilitate quality improvement and dissemination of best practices.

### Data sources

The Vizient CBD/RM contains clinical, discharge, procedure, and outcome data for each hospital encounter among member institutions. We linked the Vizient CBD/RM to annual survey data from the American Hospital Association (AHA) to ascertain institutional characteristics. [8] Institution catchment areas were defined by combining surrounding ZIP codes into Hospital Service Areas (HSAs). Socioeconomic characteristics of HSAs were gathered from the American Community Survey (ACS) through the National Historical Geographic Information System (NHGIS).[9] Specifically, we extracted demographic data by ZIP code tabulation area using ACS 5-year data for years 2008–2012.

### Study population

We examined all participating Vizient institutions contributing data to the CDB/RM between January 1, 2012, and December 31, 2012. We defined cardiac arrest as any discharge with an International Classification of Diseases, Version 9 (ICD-9) code for cardiac arrest (427.5).[10] While this approach is unable to differentiate between in-hospital cardiac arrest (IHCA) and out-of-hospital cardiac arrest (OHCA), this approach has been previously used in studies characterizing cardiac arrest epidemiology.[4, 11] In addition, we extended the definition to include discharges with an ICD-9 procedure code for cardiopulmonary resuscitation (CPR; ICD-9: 99.60) as the sole indication for CPR is cardiac arrest. We excluded patients under 18 years of age, prisoners, pregnant, under hospice care, admitted to a psychiatric unit, or transferred in from another acute care hospital. We excluded hospitals reporting fewer than twenty-five cardiac arrest discharges during the study period.

### Discharge, institution, and community characteristics

Using the Vizient CBD/RM, we identified discharge characteristics including age, gender, race, insurance type, admission to the intensive care unit (ICU), length of stay, and comorbidities. To evaluate patient comorbidities, we identified individual Elixhauser comorbidities. [12] For risk adjustment purposes, we obtained Vizient CBD/RM expected probability of mortality, which is estimated using diagnosis-related group (DRG)-specific risk models. Specifically, the risk prediction models incorporate admission mortality risk scores derived from the 3M™ APR-DRG grouper, All Patients Refined risk of mortality class (Minor, Moderate, Major, or Extreme), relevant comorbidities, admission characteristics, diagnoses, and procedures. We did not base our definition of cardiac arrest on DRG, primary diagnosis, or data suggesting cardiac arrest was present on arrival.

From the AHA survey database, we obtained institutional characteristics including hospital control, number of beds and surgical operations performed, clinical services offered, teaching hospital certification, Census region, population setting, and the number of Medicaid discharges. For teaching hospital certification, we identified institutions certified by the Accreditation Council for Graduate Medical Education (ACGME) as well as the Council of Teaching Hospitals and Health Systems (COTH) during the study period. We categorized the referral population using the National Center for Health Statistics Urban-Rural Classification Scheme, with counties classified as part of a large metropolitan area or smaller area (medium, small, or micropolitan). We defined safety net hospitals as those with Medicaid discharges that were more than one standard deviation above the national average. We assessed both total number and percentage of all discharges.

To characterize the socioeconomic status of the catchment area surrounding each hospital, we obtained the percentage living below the federal poverty level and median household income at the ZIP code level from the ACS. To better define catchment areas, we combined ZIP codes into Hospital Service Areas (HSAs), and aggregated the measures appropriately. For household income, we calculated a weighted mean based on the total population of each ZIP code within an HSA.

### Outcome measure

The primary outcome for this study was inpatient survival. We identified inpatient deaths using discharge disposition status codes available in the CDB/RM. Discharge status codes in the Vizient CBD/RM have demonstrated high concordance with results obtained via patient-level case audits. [13]

### Data analysis

For each part of our cardiac arrest definition, we calculated the percent surviving and estimated confidence intervals using the exact binomial method. We compared discharge characteristics between those who died during hospitalization and those who survived using t-tests of equal means for continuous variables and Pearson chi-square tests of association for categorical variables. To obtain risk-standardized survival rates (RSSRs) for each hospital, we fit a mixed effects logistic regression model, adjusting for Vizient CBD/RM expected mortality and specifying random intercepts. We chose to use Vizient CBD/RM expected mortality for risk adjustment, despite its proprietary nature, as it allows for a valid comparison across admitting conditions and exhibited good discrimination among cardiac arrest cases (C-statistic 0.74). Using reliability-adjusted Empirical Bayes estimates of the log odds obtained from the model, we calculated RSSRs by transforming to percentages at the average risk of mortality for the full population.[14]

We defined high performing (high survival outlier) hospitals as institutions with an RSSR significantly higher than the study cohort average (lower confidence interval limits excluding the average) and poor performing hospitals (low survival outlier) as institutions with an RSSR significantly lower than the average (upper confidence intervals excluding the average). Institutional characteristics were compared between high, low, and non-outlier survival hospitals using non-parametric Kruskal-Wallis tests of equal distribution for continuous variables, Fisher-Irwin Exact tests for categorical variables with cell sizes <5, and Pearson chi-square tests for categorical variables with cell sizes ≥5. We also examined the linear prediction of mean RSSR, modeled using volume-weighted ordinary least squares regression with restricted cubic splines over values of cardiac arrest volume (specifying 7 knots). All analyses were performed using Stata 13.1 (College Station, TX).

## Results

During 2012, the Vizient CBD/RM contained data on 3,686,296 patients admitted to 213 hospitals and a total of 33,700 (0.91%) were documented and coded cardiac arrest. (Table 1) Overall survival for all admitted patients was 97.8% (95% confidence interval (CI) 97.7–97.8) as compared to 42.3% (95% confidence interval (CI) 41.8–42.9) for those documented and coded cardiac arrest. Among those with cardiac arrest, survivors were younger and more likely to be male, Caucasian, privately insured, and have hospital stays greater than seven days. Survivors generally had fewer comorbidities compared to non-survivors, with hypertension and obesity the only comorbidities more likely among survivors. (Table 2)

**Table 1:** Cardiac arrest survival by patient type.

	Number of Visits	Inpatient Survival
	N (%)	% (95% CI)
All Visits	3,686,296	97.8 (97.7–97.8)
Any Cardiac Arrest (Diagnosis or CPR)	33,700 (0.8)	42.3 (41.8–42.9)
Arrest Diagnosis	28,954 (0.8)	44.9 (44.3–45.4)
Present on Admission	12,245 (0.3)	51.5 (50.7–52.4)
Not Present on Admission	16,971 (0.5)	39.9 (39.1–40.6)
Received CPR	15,644 (0.4)	31.4 (30.7–32.1)
Arrest Diagnosis and Received CPR	10,898 (0.3)	33.4 (32.5–34.3)

Data from 179 Vizient CDB/RM hospitals in 2012. Excludes hospitals with fewer than 25 eligible cardiac arrest cases. Excluding patients aged <18 years, prisoners, transfers, and hospice patients.

**Table 2:** Cardiac arrest patient characteristics by inpatient mortality.

	Died	Survived	p
	N (%)	N (%)	
	19,435	14,265	
Age (Years) (Mean; SD)*	63.8 (17.1)	61.1 (15.8)	<0.001
Age (Years)*			<0.001
18–40	1,993 (10.3)	1,565 (11.0)	
41–60	5,633 (29.0)	4,939 (34.6)	
61–80	8,301 (42.8)	6,246 (43.8)	
81+	3,485 (18.0)	1,513 (10.6)	
Gender**			<0.001
Male	11,254 (57.9)	8,875 (62.2)	
Female	8,177 (42.1)	5,390 (37.8)	
Race***			<0.001
White/Caucasian	11,513 (60.8)	9,330 (66.5)	
Black/African American	4,964 (26.2)	3,201 (22.8)	
Other	2,472 (13.1)	1,507 (10.7)	
Pay type			<0.001
Medicare	11,811 (60.8)	7,795 (54.6)	
Medicaid	2,747 (14.1)	1,941 (13.6)	
Private Insurance	3,027 (15.6)	3,472 (24.3)	
Self-Pay	1,365 (7.0)	674 (4.7)	
Other	485 (2.5)	383 (2.7)	
Pay type (among 17,494 aged <65 yrs)			<0.001
Medicare	2,566 (27.1)	2,022 (25.2)	
Medicaid	2,522 (26.6)	1,828 (22.8)	
Private Insurance	2,731 (28.8)	3,202 (39.9)	
Self-Pay	1,244 (13.1)	634 (7.9)	
Other	410 (4.3)	335 (4.2)	
ICU During Admission†			<0.001
Yes	14,623 (76.6)	11,342 (80.9)	
No	4,461 (23.4)	2,677 (19.1)	
Length of Stay (days)‡			<0.001
≤ 2	8,047 (42.3)	1,455 (10.8)	
3–6	4,488 (23.6)	3,261 (24.1)	
≥ 7	6,509 (34.2)	8,812 (65.1)	
Weighted Charlson Comorbidity Score			<0.001
0	2,693 (13.9)	2,037 (14.3)	
1	3,151 (16.2)	3,134 (22.0)	
≥ 2	13,591 (69.9)	9,094 (63.8)	
Elixhauser Comorbidity Group			
Valvular Disease	1,650 (8.5)	855 (6.0)	<0.001
Congestive Heart Failure	3,816 (19.6)	2,072 (14.5)	<0.001
Pulmonary Circulation Disease	1,675 (8.6)	755 (5.3)	<0.001
Chronic Pulmonary Disease	4,388 (22.6)	3,007 (21.1)	0.002
Hypertension	10,217 (52.6)	7,785 (54.6)	<0.001
Diabetes	6,429 (33.1)	4,629 (32.5)	0.147
Renal Failure	5,714 (29.4)	3,740 (26.2)	<0.001
Liver Disease	1,500 (7.7)	633 (4.4)	<0.001
Metastatic Cancer	1,038 (5.3)	261 (1.8)	<0.001
Solid Tumor	695 (3.6)	276 (1.9)	<0.001
Coagulopathy	3,274 (16.9)	1,350 (9.5)	<0.001
Anemia	5,156 (26.5)	3,445 (24.2)	<0.001
Obesity	2,622 (13.5)	2,514 (17.6)	<0.001

Data from 179 Vizient CDB/RM hospitals in 2012. Excludes hospitals with fewer than 25 eligible cardiac arrest cases.

* 25 missing age.

** 4 missing gender.

*** 713 missing race.

† 597 missing ICU admission.

‡ 1,128 missing length of stay. Excluding those aged <18 years, prisoners, transfers, and hospice patients. P-values from Pearson chi-square tests of association.

Among 179 institutions reporting more than 25 cardiac arrest patients, there was wide regional variation in the number of cardiac arrest cases (Table 3), variation in the hospital volume (median 162; IQR 91–250, Table 4), and observed survival (40.5; 34.6–46.6), and RSSRs (38.2; 34.1–42.5). (Table 5) After adjusting for expected mortality, the intraclass correlation among study hospitals was 0.032 (95% CI 0.024–0.043), suggesting substantial variation in survival across institutions.

**Table 3:** Cardiac arrest by geographic region.

	Arrest Diagnosis	Received CPR
	N (%)	N (%)
**Census Region**		
Northeast (N = 9,074)	7,822 (86.2)	4,060 (44.7)
Midwest (N = 8,553)	7,406 (86.6)	4,305 (50.3)
South (N = 11,509)	9,962 (86.6)	5,151 (44.8)
West (N = 4,564)	4,026 (88.2)	2,128 (46.6)

Data from 179 Vizient CDB/RM hospitals in 2012, including 33,700 discharges with cardiac arrest. Excludes hospitals with fewer than 25 eligible cardiac arrest cases. Excluding patients aged <18 years, prisoners, transfers, and hospice patients. P-value for Arrest Diagnosis = <0.001, CPR = <0.001.

**Table 4:** Characteristics of Vizient CDB/RM hospitals, 2012.

	Median (IQR)	Min	10^th^ Pct	90^th^ Pct	Max
Total Patients	22,869 (12,952–32,892)	1,817	7,942	48,325	112,379
Cardiac Arrest Cases	162 (91–250)	25	39	369	768
Number of Cardiac Arrest Patients Surviving	66 (29–108)	6	14	160	364
Observed Cardiac Arrest Survival (%)	40.5 (34.6–46.6)	17.1	28.0	52.3	74.1
RSSR (%)	38.2 (34.1–42.5)	22.0	29.7	46.2	59.6

N = 179 Vizient CDB/RM hospitals. Excludes hospitals with fewer than 25 eligible cardiac arrest cases. Excluding those aged <18 years, prisoners, transfers, and hospice patients. IQR = interquartile range, RSSR = risk-standardized survival rate. RSSRs estimated using Empirical Bayes log odds estimates obtained from a random intercept logistic regression model adjusted for expected mortality. All RSSR estimates were transformed to percentages and are provided for discharges at the average expected mortality value (probability of mortality = 0.24).

**Table 5:** Institutional characteristics by risk-standardized survival rate performance.

Hospital Characteristic	RSSR Performance			p
	Average; 29.9–45.7% (N = 131)	Low; 22.0–32.5% (N = 20)	High; 43.3–59.6% (N = 28)	
CA Volume (Median, IQR)	133 (62–233)	119 (95–223)	262 (159–376)	<0.001
CA Volume per 10k Disch (Median, IQR)	83.8 (65.0–105.5)	85.2 (71.3–126.1)	91.1 (78.9–113.4)	0.19
CA Volume per 10 Beds (Median, IQR)	3.6 (2.9–4.7)	3.7 (2.8–4.4)	4.4 (3.0–5.2)	0.20
Hospital Control (N, %)				0.19
Government, Non-Federal	33 (25.2)	10 (50.0)	7 (25.0)	
Non-Government, Not-for-Profit	93 (71.0)	10 (50.0)	21 (75.0)	
Investor-Owned	5 (3.8)	0 (0)	0 (0)	
Total Number of Beds (Median, IQR)	396 (210–596)	452 (277–559)	722 (447–839)	<0.001
Services Offered (N, %)				
Cardiac Catheterization*	106 (84.8)	13 (72.2)	27 (100.0)	0.03
Cardiac Surgery*	91 (72.8)	11 (61.1)	25 (92.6)	0.04
Transplant*	68 (54.4)	11 (61.1)	23 (85.2)	0.01
Neurological*	121 (96.8)	18 (100.0)	27 (100.0)	—
Oncology*	122 (97.6)	18 (100.0)	27 (100.0)	—
Trauma*	74 (59.2)	11 (61.1)	24 (88.9)	0.01
Therapeutic Hypothermia	128 (97.7)	20 (100.0)	28 (100.0)	—
Annual Number of Surgical Operations (Median, IQR)	13,954 (7,945–22,184)	13,109 (9,793–18,611)	24,939 (16,661–38,661)	<0.001
Hospital Teaching Certification (N, %)				
ACGME	97 (74.1)	16 (80.0)	25 (89.3)	0.22†
COTH	79 (60.3)	14 (70.0)	25 (89.3)	0.01
Census Region (N, %)				0.49†
Northeast	36 (27.5)	**	11 (39.3)	
Midwest	37 (28.2)	**	7 (25.0)	
South	41 (31.3)	9 (45.0)	5 (17.9)	
West	17 (13.0)	**	5 (17.9)	
Population Setting (N, %)				0.29
Large Metropolitan Area	94 (71.8)	14 (70.0)	**	
Micro/Small/Med Metropolitan Area	37 (28.2)	6 (30.0)	**	
Population Socioeconomic Profile				
Percentage Medicaid Discharges (Median, IQR)€	19.6 (13.7–25.6)	29.7 (17.5–37.8)	21.9 (15.8–29.1)	0.01
Safety Net (Num of Disch) (N, %)€	67 (51.5)	12 (60.0)	23 (82.1)	0.01
Safety Net (Perc Medicaid) (N, %)€	27 (20.8)	9 (45.0)	7 (25.0)	0.06
HSA Median Household Income (Median, IQR)	58,363 (50,140–68,476)	49,104 (45,681–60,516)	61,922 (58,255–67,414)	0.02
HSA % in Poverty (Median, IQR)	15.1 (11.0–17.8)	17.3 (15.5–22.5)	14.6 (11.6–17.8)	0.03

N = 179 Vizient CDB/RM hospitals. Excludes hospitals with fewer than 25 eligible cardiac arrest cases. Excluding those aged <18 years, prisoners, transfers, and hospice patients.

* 9 hospitals missing data.

** Suppressed due to count <5.

€ 1 hospital missing data.

P-values from Kruskal-Wallis tests of equal distribution for continuous variables and Pearson Chi-Square tests of association or Fisher’s Exact tests (†) for categorical variables.

RSSR = risk-standardized survival rate; IQR = interquartile range (25^th^-75^th^ percentiles); COTH = member of Council of Teaching Hospital of the Association of American Medical Colleges; ACGME = residency training approval by Accreditation Council for Graduate Medical Education; HSA = hospital service area.

**Fig 1:**
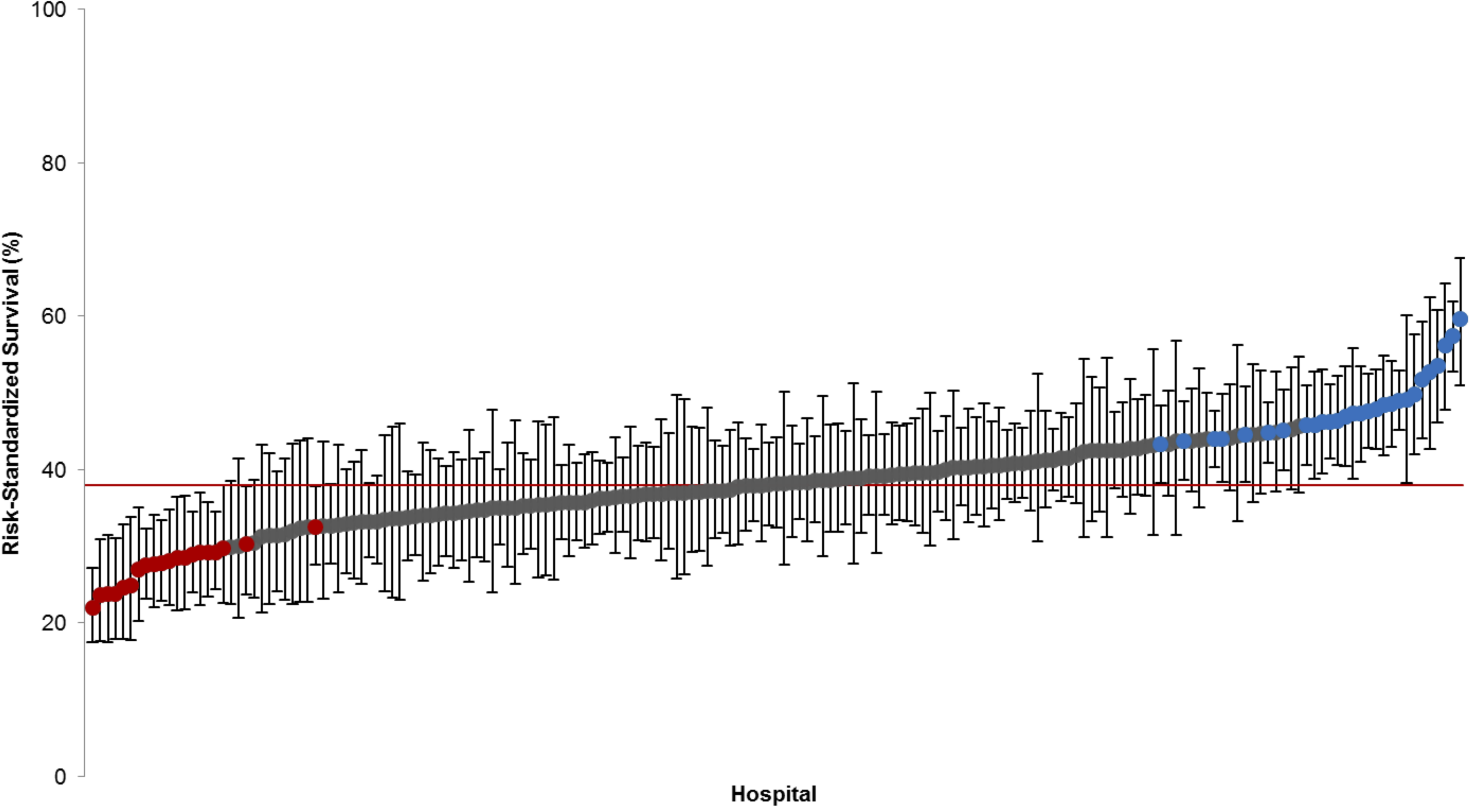
Institutional variation in risk-standardized survival rate. N = 179 Vizient CDB/RM hospitals. RSSRs estimated using Empirical Bayes log odds estimates obtained from a random intercept logistic regression model adjusted for expected mortality. All RSSR estimates were transformed to percentages and are provided for discharges at the average expected mortality value (probability of mortality = 0.24). Markers indicate hospitals performing significantly worse (Red, N = 20) or better (Blue, N = 28) than the average. Red reference line indicates overall population survival at the average expected mortality (38.0%). Error bars represent 95% confidence interval limits.

**Fig 2:**
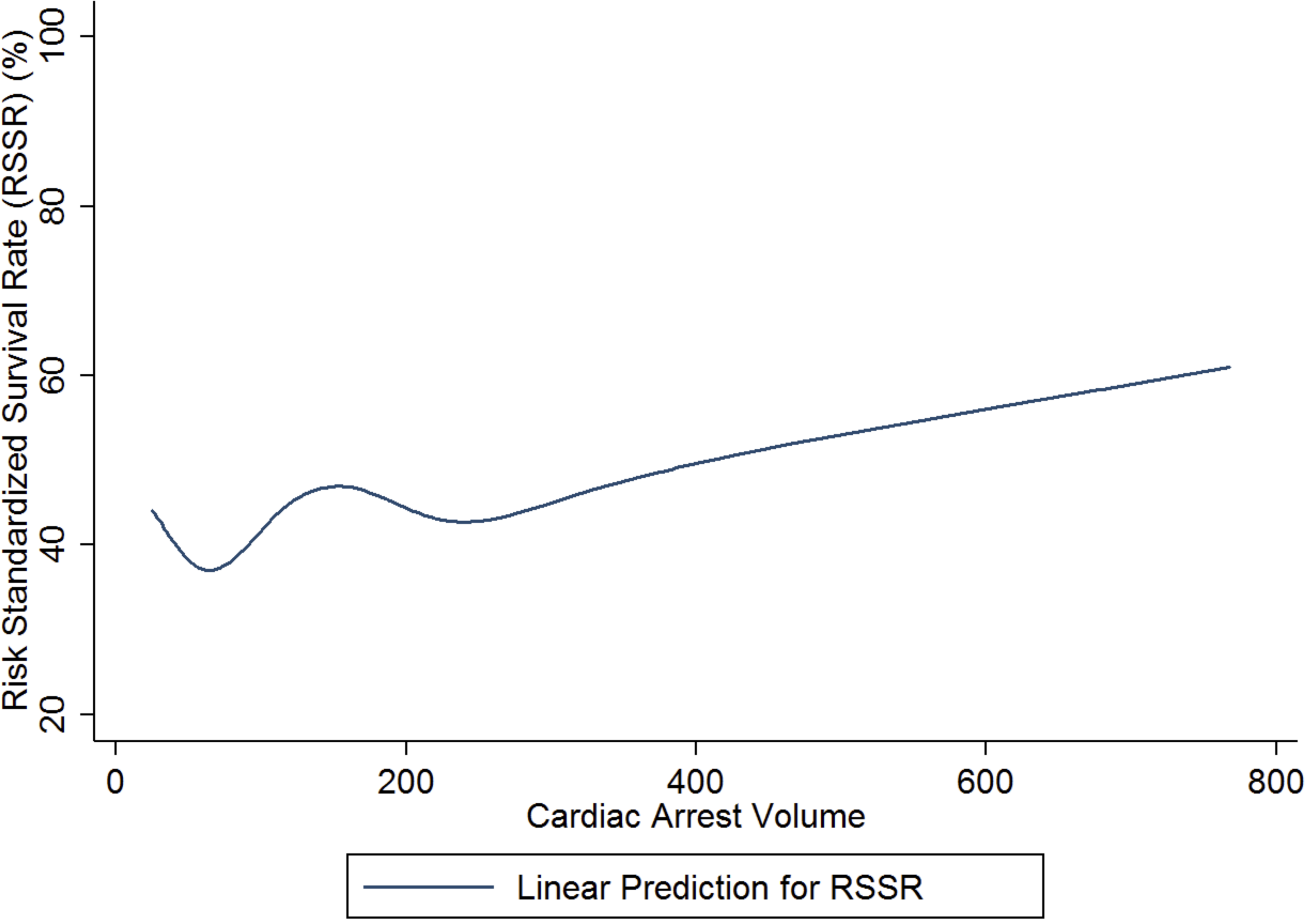
Risk standardized survival rate as a function of hospital volume. N = 179 Vizient CDB/RM hospitals. RSSRs estimated using Empirical Bayes log odds estimates obtained from a random intercept logistic regression model adjusted for expected mortality. All RSSR estimates were transformed to percentages and are provided for discharges at the average expected mortality value (probability of mortality = 0.24). Linear prediction represents the predicted mean RSSR modeled using volume-weighted ordinary least squares regression with restricted cubic splines over values of cardiac arrest volume.

There were 28 hospitals (15.6%) with higher than average survival (median RSSR 47.3%; interquartile range (IQR) 45.4–49.5; Min-Max 43.3–59.6) and 20 hospitals (11.2%) with lower than average survival (median RSSR 28.0%; IQR 24.8–29.2; Min-Max 29.9–45.7). (Fig 1) Compared to hospitals with lower than average cardiac arrest survival, those with high survival had higher cardiac arrest volume, a greater number of beds, higher annual surgical volume, and were more likely to be teaching hospitals and offer cardiac catheterization, cardiac surgery, trauma, and transplant services. (Fig 2) High survival hospitals also had a lower percentage of Medicaid discharges and surrounding HSA catchment areas that consisted of populations with higher median household income and lower percentage living in poverty. (Table 5)

## Discussion

In this analysis of hospitals affiliated with academic medical centers, we observed wide variations in cardiac arrest survival, even after accounting for differences in expected mortality. Institutions with higher risk-standardized cardiac arrest survival were larger, had larger cardiac arrest and surgical case volume, higher catchment area socioeconomic status, and greater availability of trauma and cardiac surgery services. Multiple factors support the external validity of these findings. Our analysis is derived from the Vizient CDB/RM, encompassing more than 3.5 million hospital admissions across 179 academic institutions in 2012, or approximately 10% of annual hospital admissions in the United States. Our findings are novel as they represent the most recent observational, risk-adjusted assessment of hospital level operational characteristics that contribute to improved cardiac arrest survival.

The identification of hospital-level factors associated with cardiac arrest outcomes suggests that there may be variation in the care of cardiac arrest patients. For the first time in 2015, the evidenced-based AHA guidelines for emergency cardiac care include recommendations for systems of resuscitation care for cardiac arrest, regardless of location.[6] Some of those criteria, such as the availability of percutaneous coronary intervention (PCI), are supported by our investigation. Our data did not have information to evaluate other guideline recommended response elements such as appropriate institutional surveillance (i.e. early warning system), dedicated in-hospital response, (i.e. rapid response team), and availability of a comprehensive, post cardiac arrest service.

Our results support previous epidemiological studies suggesting higher cardiac arrest survival among large, teaching institutions [4] [5] that care for high cardiac arrest volume. [15] The association between case volume and outcome has previously been demonstrated in other critical illnesses such as stroke,[16] trauma,[17] and myocardial infarction[18] where quality treatment is similarly time-dependent. However, unlike these previous studies, our analysis demonstrated specific hospital offerings, for example, cardiac catheterization and cardiac surgery, and higher annual surgery volume were associated with higher than average survival. While previous meta-analysis has demonstrated an association between cardiac arrest survival and PCI, [19] the characteristics identified by our results suggest that these hospital offerings may serve as surrogates for a concentration of expertise in caring for the critically ill.

A number of experts have drawn a clear association between lower socioeconomic status, predominately minority, neighborhoods and survival from cardiac arrest. [20] Previously those associations have been linked to pre-hospital factors such as 9-1-1 access and bystander CPR rates. [21] While such pre-hospital factors clearly impact survival to hospital discharge from cardiac arrest, our investigation is novel in that it demonstrates an association between the socioeconomic status of the hospital catchment area and cardiac arrest outcomes. These findings suggest that some portion of the geographic variation in survival from cardiac arrest may reflect characteristics of the institutional referral base in addition to the quality of the treatment delivered or the systems of care employed.

Our investigation in academic center-affiliated hospitals identified wide variation in cardiac arrest survival. While such variation in survival has previously been demonstrated, [4] our investigation quantified a three-fold difference in survival between best and weakest performing study hospitals suggesting opportunities to improve cardiac arrest care. Further research should be directed to exploring causes for the institutional variations we observed and clarifying the role of the institutional factors in cardiac arrest survival performance. If survival can be causally linked to hospital characteristics, then mechanisms for developing systems of care to reduce geographic variation and optimize care for all victims of cardiac arrest are achievable.

## Limitations

Our observations derive from data submitted by hospitals affiliated with academic medical centers, some of which have mature systems of care to maximize cardiac arrest outcomes. As such, survival rates may differ for non-academic centers. We excluded transfers from outside hospitals, as the reason for transfer could not be determined from the data, and therefore this “non-native” population could have impacted the associations identified between risk factors and survival.

The greatest limitation of this investigation arises from the use of discharge diagnoses for case-definition and outcome measures. Reliance on such administrative classification assumes concordance between medical coding technicians and treating clinicians. While this strategy may lead to significant under-classification as suggested by Coppler et. al.,[22] it has been used in previous epidemiologic studies examining cardiac arrest [4] [11] and other complex conditions. [23] [24] Furthermore, our analysis is strengthened by the novel use of the ICD-9 procedure code for CPR (ICD-9: 99.60) as inclusion criteria, which yielded 4,746 (16.4%) additional subjects.

We did not have access to initial cardiac rhythm, timing of defibrillation (when appropriate), duration and quality of CPR, or other Utstein factors previously demonstrated to impact survival. [25] While the Vizient CDB/RM risk models provide a comprehensive strategy for risk adjustment, our estimated RSSRs did not reflect traditionally collected Utstein factors that may provide improved model discrimination. In addition, these data do not allow for further description of neurologic status upon discharge, a more meaningful qualifier of treatment success beyond mere survival.

## Conclusion

Among study cohort hospitals, there was wide variation in cardiac arrest survival and institutions with higher cardiac arrest and surgical case volume, availability of catheterization and cardiac surgery services, and higher catchment area socioeconomic status demonstrated higher risk-standardized survival. Further efforts should seek to examine these observations in greater detail and identify specific strategies for improving outcomes among hospitals with low cardiac arrest survival.
